# Management of Familial Hypercholesterolemia with Special Emphasis on Evinacumab

**DOI:** 10.3390/biomedicines10123273

**Published:** 2022-12-16

**Authors:** Julia Krzemińska, Ewelina Młynarska, Ewa Radzioch, Magdalena Wronka, Jacek Rysz, Beata Franczyk

**Affiliations:** 1 Department of Nephrocardiology, Medical University of Lodz, ul. Zeromskiego 113, 90-549 Lodz, Poland; 2 Department of Nephrology, Hypertension and Family Medicine, Medical University of Lodz, ul. Zeromskiego 113, 90-549 Lodz, Poland

**Keywords:** cardiovascular risk, familial hypercholesterolemia (FH), evinacumab, ANGPTL3, hypolipidemic therapy

## Abstract

Familial hypercholesterolemia (FH) is an underdiagnosed disease that contributes to a significant number of cardiovascular incidents through high serum Low-Density Lipoprotein Cholesterol (LDL-C) values. Its treatment primarily requires healthy lifestyle and therapy based on statins, ezetimibe and Proprotein Convertase Subtilisin/Kexin Type 9 (PCSK9); however, there are also new treatment options that can be used in patients who do not respond to therapy, among which we highlight evinacumab. Elevated LDL-C values, together with clinical manifestations associated with cholesterol deposition (e.g., tendon xanthomas, xanthelasma and arcus cornealis) and family history are the main elements in the diagnosis of FH. Pathognomonic signs of FH include extensor tendon xanthomas; however, their absence does not exclude the diagnosis. Elevated LDL-C levels lead to premature Atherosclerotic Cardiovascular Disease (ASCVD), which is why early diagnosis and treatment of FH is essential. Evinacumab, a novelty in pharmacological practice, having a complex mechanism of action, causes desirable changes in lipid parameters in patients with homozygous form of familial hypercholesterolemia (HoFH). This review collects and summarizes the most important aspects of the new drug, especially being a discovery in the treatment of HoFH, giving these patients hope for a longer and more comfortable life.

## 1. Introduction

**Figure 1:**
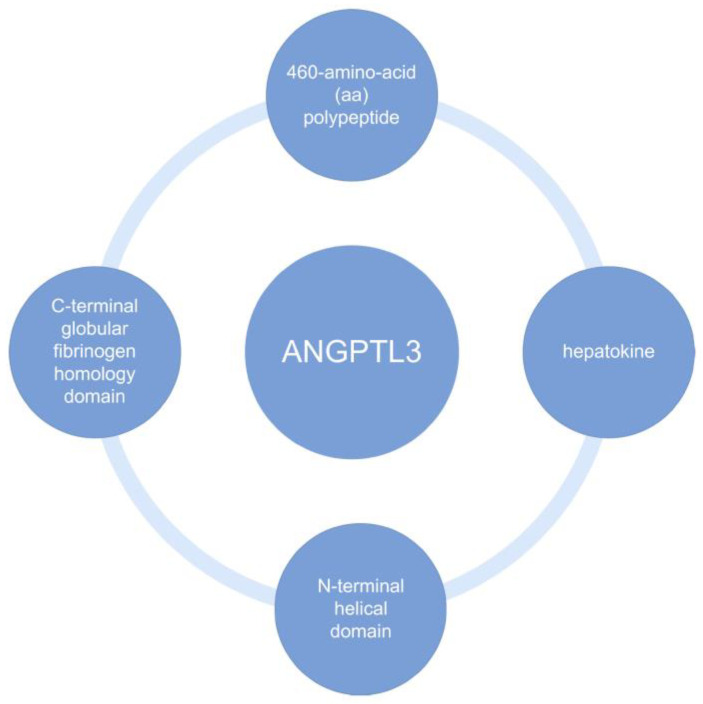
Biochemical Structure of ANGPTL3 [21,22]. ANGPTL3, angiopoietin-like protein 3.

Familial hypercholesterolemia (FH) is an inherited disorder characterized by very high levels of Low-Density Lipoprotein Cholesterol (LDL-C)—Exceeding the upper limit of normal [1]. Clinical manifestation of FH depends on its form—Heterozygous or homozygous [2,3]. The heterozygous familial hypercholesterolemia (HeFH) form is characterized by LDL-C concentrations > 190 mg/dL [2], later onset of symptoms [4], while clinical manifestations are usually limited to extensor tendon xanthomas [5]. On the contrary, in homozygous familial hypercholesterolemia (HoFH), LDL-C concentrations can reach >500 mg/dL [3] and the first symptoms appear as early as childhood [4]. Due to the risk of premature onset of atherosclerotic cardiovascular disease (ASCVD) [2,3,6], it is very important to diagnose FH early [4]. A diagnosis is mainly based on the assessment of LDL-C levels, clinical symptoms, family history [7] and is made using appropriate criteria [8,9], which are presented in this paper. Patients with FH have an increased cardiovascular risk because the disease leads to increased and premature atherosclerosis, primarily the deposition of atherosclerotic plaques in the coronary arteries with subsequent complications, such as myocardial infarction (MI) [10,11]. An important role in the treatment of people with FH is to maintain a healthy lifestyle and immediately initiate intensive hypolipidemic pharmacotherapy [1,10]. Patients should follow an appropriate diet rich in fruits and vegetables, regular physical activity and, if overweight or obese, aim to reduce excess body weight [1]. Pharmacotherapy is primarily based on statins, however, in most cases, it is necessary to intensify it by including ezetimibe and Proprotein Convertase Subtilisin Kexin Type 9 (PCSK9) inhibitors [12]. Evinacumab—a drug developed by Regeneron Pharmaceuticals and registered with the Food and Drug Administration (FDA) in February 2021, with European Union approval—is an IgG4 monoclonal antibody against angiopoietin-like 3 protein (ANGPTL3) [13,14,15,16]. ANGPTL3 is a protein containing polysaccharide chains which is endogenously produced in the liver and kidney [17,18]. It is one of eight ANGPTL1–ANGPTL8 proteins related to vascular endothelial growth factor [15,16,18]. ANGPTL3 is responsible for the metabolism of lipids found in plasma, being an inhibitor of both lipoprotein lipase (LPL), endothelial lipase (EL) and hepatic lipase (HL) [14,19,20]. Biochemical structures of ANGPTL3 are shown in the Figure 1 below [21,22].

## 2. Basic Information about FH and Its Genetic Background

FH is a relatively common hereditary condition found worldwide with a usually autosomal dominant mode of inheritance, characterized by very high serum concentrations of the LDL-C fraction—exceeding the upper limit of normal [1,10]. In the United States, the prevalence of this disease is estimated at 1:250 people aged >20 years [23]. In the lipidogram of patients in FH, in addition to increased levels of LDL-C, there is a subsequent increase in total cholesterol, normal or reduced levels of High-Density Lipoprotein (HDL) and usually normal levels of triglycerides (TG) [10]. In addition, higher levels of lipoprotein a (Lp(a)) are also present, which further increases the high risk of coronary artery disease (CAD) [12].

The pathogenesis of FH is complex and involves multiple genetic variants. The gene responsible for the manifestation of FH is located on chromosome 19 [10,23]. Due to the mode of inheritance of genetic disorders of FH, it can be divided into autosomal dominant HoFH, autosomal recessive HeFH and polygenic hypercholesterolemia [12]. It is mostly caused by monogenic mutations that cause a defect in the Low-Density Lipoprotein Receptor (LDLR), thereby disrupting the uptake of Low Density Lipoprotein (LDL) by hepatocyte cells [24]. Both offspring and siblings of patients have a 50% chance of inheriting the condition [25]. HoFH involves a mutation inherited from both parents. If the mutation inherited from two parents involves the same gene, it is truly HoFH. However, it is also possible to inherit a different mutation in the LDLR gene from each parent, which is called compound heterozygote [12]. However, both situations lead to a similar clinical picture with very high LDL-C levels [1]. However, the homozygous variant of FH is much less common and it is associated with a severe form of the disease [10,23]. Additionally, it is noteworthy that, despite the difficulty of genetic diagnosis, patients with very high LDL-C levels, exceeding 310 mg/dL, are 92% carriers of the monogenic homozygous mutation. However, a common variant of FH is a form involving mutations affecting multiple genes. It has been suggested that up to 50% of patients, in whom hypolipidemic treatment is implemented and a genetic defect is suspected in the form of FH, have multigene hypercholesterolemia. It is also worth noting that patients with this variant of the disease can often have a negative genotype. Heterozygous form of FH, on the other hand, is a very rare variant. Despite everything, regardless of the form, there is an increase in LDL-C in every case [12].

There are many types of mutations involved in the development of HoFH and the three most common and major mutations that lead to the development of FH are defects in LDLR, PCSK9, and Apolipoprotein B (APOB). The most common of these—the classic mutation causing 90% of ADH—are mutations in the LDLR gene. Non-classical FH includes mutations in the APOB and PCSK9 genes, which cause similar symptoms and severity of the disease as in the classical form. Table 1 shows and describes the main mutations involved in the development of HoFH [12,23]. In addition to these most common mutations causing hypercholesterolemia, there are other rare recessive mutations, one of which is a homozygous mutation of the CYP7A1 gene, also leading to high serum LDL-C concentrations [12]. Regardless of the type of mutation, in each case the LDL-C receptors are disrupted, so that hepatic uptake of LDL-C is impaired and high serum LDL-C concentrations are observed [23]. Sometimes it is difficult to identify the specific defect that led to the severe form of FH. Often the disease is caused by a multigene mutation, thus the identification of a genetic variant is not a necessary criterion for the diagnosis of FH [12].

## 3. Clinical Manifestations of FH

FH manifests clinically with high levels of circulating LDL-C [2,3,6,26], premature onset of ASCVD [2,3,6], and the accumulation of cholesterol deposits in tissues and/or organs—mainly tendon xanthomas [3,5,6,7,8,9,26]. Symptoms occur in both the patient and his relatives [2,6].

Clinical manifestation, including LDL-C values, depends on the form of FH [2,3]. HeFH is characterized by LDL-C values > 190 mg/dL in adults, and >160 mg/dL in children [2]. While in HoFH, LDL-C levels reach as high as >500 mg/dL [3]. High levels of LDL-C lead to premature ASCVD [2,3], which will be described in detail in paragraph six.

**Figure 2:**
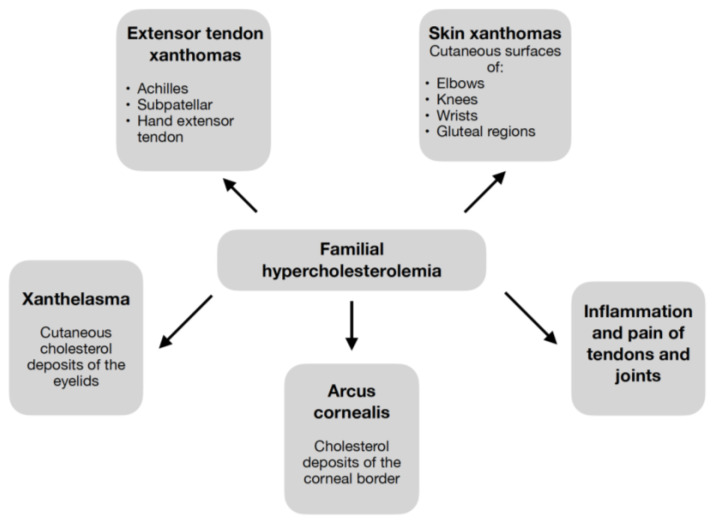
Symptoms Associated with Cholesterol Deposition in the Course of FH [2,3,5,6,7,8,9,26].

Elevated LDL-C values contribute to the symptoms of cholesterol deposition shown in Figure 2 [2,3,5,6,7,8,9,26]. Pathognomonic for FH are xanthomas—cholesterol deposits in the extensor tendons [3,7,26], which, regardless of the patient’s age, should always raise suspicion of FH [9].

Symptoms appear as early as childhood in HoFH [4] and can include a wide range of clinical manifestations (Figure 2) [3,5,9]. While in HeFH it occurs much later [4], usually after several decades, and the lesions are often limited to the xanthomas of the tendons [5]. Detection of xanthomas, in addition to directing to the diagnosis of FH, carries important information about the extent of atherosclerosis and the patient’s cardiovascular risk [5,26,27].

## 4. Diagnosis of FH

HeFH is usually diagnosed incidentally during routine blood tests, often in the adolescent or young adult years. At that time, elevated LDL-C levels are usually the only one sign of the disease. However, sometimes the diagnosis of HeFH is made after a patient experiences premature ASCVD [8]. The situation is different with HoFH, where the first symptoms occur as early as childhood [3,5,9]. Failure to diagnose and treat HoFH leads to death in patients < 20 years old [5,9].

The diagnosis of HeFH is usually based on one of several diagnostic criteria [8,9]: Dutch Lipid Clinic Network (DLCN) [6,7,8,9,26,28,29,30], Simon Broome Criteria [6,7,8,26,28,29,30], Make Early Diagnosis to Prevent Early Death (MEDPED) [7,26,28,30,31], and the Familial Hypercholesterolemia Case Ascertainment Tool (FAMCAT) [8,32], the International Statistical Classification of Diseases and Related Health Problems, 10th Revision (ICD-10) diagnostic criteria [8], National Lipid Association recommendations [9,26], and others. The most common and extensive diagnostic criteria for FH [7,28,30] are DLCN [6,7,8,9,26,28,29,30], Simon Broome Criteria [6,7,8,26,28,29,30], and MEDPED [7,26,28,30,31]. Of these, none is superior to the others. What they have in common is an assessment of LDL-C levels, a history of FH-specific clinical manifestations, and a family history [7].

The DLCN criteria are claimed to provide the most accurate assessment of the presence of HeFH [26] and are the most widely used criteria for diagnosing FH [7]. However, they cannot be applied to children and adolescents [5,9]. The diagnostic criteria for DLCN have been approved by the European Atherosclerosis Society (EAS) [8].

Simon Broome along with the DLCN are the main criteria for the diagnosis of FH [6,8,29]. The difference is that in the Simon Broome criteria, unlike the DLCN criteria, only the mutation present in the LDL-R, ApoB, or PCSK9 gene is sufficient for the diagnosis of FH [26]. In addition, clinical manifestation in the form of arcus cornealis is not considered [9]. The Simon Broome criteria are also applicable in children and adolescents [5,9] with the LDL-C cutoff point for children differing from that proposed for adults and being >160 mg/dL [5].

MEDPED criteria take into account an elevated LDL-C level and a positive family history of hypercholesterolemia [7,26]. Shah et al. [7] reports that the sensitivity of MEDPED for the diagnosis of HeFH is 54% and the specificity is 98%. The sensitivity of MEDPED varies with the degree of consanguinity of the relative suffering from HeFH. It is highest (88%) for patients with a first-degree relative suffering from HeFH. These reports are consistent with those of Hopkins et al. [31], who highlights the high sensitivity (>95%) and specificity of MEDPED. The high accuracy of the described criterion may effectively increase the diagnosis of FH.

FAMCAT is a criterion that, unlike others, takes into account, e.g., statin administration, TG levels, or chronic kidney disease [32]. Studies in the UK have shown that it is a sensitive and useful criterion for detecting FH; however, studies in other countries and international validation of this tool are needed [8,32].

The ICD-10 diagnostic criteria for HeFH have been approved by the American Heart Association, and they show greater sensitivity than the DLCN criteria [8].

According to NLA recommendations, LDL-C values ≥ 160 mg/dL or HDL values ≥190 mg/dL in patients < 20 years old suggest FH [9]. Importantly, the NLA statement supports the use of diagnostic criteria, such as DLCN, Simon Broome, and MEDPED [26].

The diagnostic criteria described above cannot be applied to the diagnosis of HoFH. Suspicion of HoFH should be aroused by clinical manifestations, such as LDL-C levels > 500 mg/dL [7], however, lower values do not disqualify the diagnosis [33], xanthomas of the extensor tendons, especially xanthomas of the skin developing in childhood [3,7], or a positive family history [7].

The described clinical criteria are summarized in Table 2 [7,8,26,32,33,34].

When diagnosing FH, it is important to consider whether the patient has other conditions that can lead to elevated LDL-C values. Such conditions will include many chronic diseases, e.g., hypothyroidism [2,5,8,9,26,29,35], liver dysfunction [5,8,26,29,35], nephrotic syndrome [2,5,8,9,29], significant proteinuria [35], and diabetes [29]. In addition, the use of drugs, such as amiodarone, cyclosporine [26], corticosteroids [29], and diuretics (hydrochlorothiazide, chlorthalidone) [26,29], which can also lead to dyslipidemia, should be excluded [5,26,29]. False high LDL-C results are also obtained by patients with high levels of Lp(a) [8,35], in which cholesterol accounts for 20–30% by weight, which is the same as in LDL-C. When calculating LDL-C levels based on the Friedewald formula, Lp(a) and LDL-C are counted together as LDL-C, inflating the result [8].

FH is a disease entity that often goes unrecognized in patients suffering from it [2,4,9,26,29,32,35]. It is estimated that FH has been diagnosed in only 15% of patients in the UK [32], and <10% of patients suffering from FH in the United States [2]. There are many reasons for the underdiagnosis of this disease; among them, socioeconomic factors and lack of knowledge of FH epidemiology play an important role [32]. Early diagnosis of FH is important because of the increased cardiovascular risk and the need for prompt initiation of therapy [4,8]. Such action makes it possible to prevent premature ASCVD in patients with FH [2,35]. Primary care physicians [26] and laboratory personnel can contribute to improving the recognition of FH. It is proposed that if a high LDL-C result is obtained on laboratory tests, the laboratory report should include a note that the patient may have FH. Such solutions will help to increase the percentage of correctly diagnosed patients suffering from FH [2,35].

## 5. Treatment of FH

The aim of therapy for patients with genetic hypercholesterolemia is to prevent cardiovascular events by aggressively reducing lipid levels with a healthy, balanced diet, physical activity, and hypolipidemic pharmacotherapy [12]. An important role in the treatment of people with FH is to maintain a healthy lifestyle. Patients should have an adequate diet rich in fruits and vegetables, regular physical activity, control body weight and, if overweight or obese, aim to reduce excess body weight, limit alcohol consumption, and stop smoking [1,23]. In the treatment process, it is also important that patients and their families are informed of the need for pharmacotherapy for the rest of their lives [23]. Aggressive lowering of serum LDL-C levels is important in FH therapy because failure to treat can increase the risk of death from aggravated ASCVD by up to 90 times [10]. According to the guidelines, hypolipemic treatment should be implemented at LDL-C levels above 190 mg/dL aiming to reduce its level by at least 50% from the pre-treatment value or <100 mg/dL in adults or <70 mg/dL in those with known diabetes/CAD. It is also recommended to reduce LDL-C to <55 mg/dL in patients with ASCVD and additional risk factors, e.g., diabetes and acute coronary syndrome (ACS) [12,23]. If FH was diagnosed in childhood, patients should be given special care by recommending a health-promoting lifestyle, implementing statin-based pharmacotherapy to effectively reduce serum LDL-C levels and prevent premature cardiovascular events in adulthood [10]. Genetic testing can be helpful in facilitating diagnosis, selecting appropriately intensified treatment depending on the mutation, however it is not mandatory [12].

Pharmacotherapy for patients with FH is primarily based on statins, which are first-line drugs [12,23,25]. These patients should be treated with intensive statin therapy from the beginning, choosing high-intensity ones, as they significantly reduce the risk of non-fatal MI [1,25]. During therapy, the goal should be to reduce LDL-C levels by at least 50% from pretreatment values. The statin dose should be increased to the maximum or as high as possible tolerated by the patient [1]. Unfortunately, despite maximally tolerated doses and 50% reduction in LDL-C, patients often do not achieve the targeted LDL-C level because their initial levels are too high; thus, it is necessary to intensify therapy by adding ezetimibe and/or PCSK9 inhibitors [12,23]. Ezetimibe is one of the second-line drugs, while PCSK9 inhibitors are second- or third-line drugs [23]. If combination therapy with the maximum possible dose of a high-intensity statin plus ezetimibe does not lead to a reduction in LDL levels of at least 50% compared to pretreatment values, or the patient has a high risk of a “cardiac incident”, the patient should be referred to an experienced specialist [1]. Therapy based on fibrates, bile acid sequestrant, or nicotinic acid is of little use in FH as the available above drugs are more effective [25]. Another therapeutic option is to include bempedoic acid, as biochemically is a precursor to the drug, as its activation occurs in the liver causing the formation of bempedoyl-CoA. In studies, the drug showed not only a reduction in LDL levels, but also in other atherogenic lipoproteins, and caused a marked reduction in factors responsible for inflammation [36]. Mipomersen is a drug which is an antisense oligonucleotide. Its mechanism of action is based on the reduction of apoB-100 synthesis in the liver. Mipomersen lowers plasma concentrations of total cholesterol (TC), LDL-C, non-high-density lipoprotein (non-HDL-C), and apoB, and Lp(a) [37] in an LDL-R-independent mechanism [38]. A study by Duell et al. [37] showed that its long-term use in FH patients effectively reduces the risk of cardiovascular diseases (CVD) events. In addition, it is worth focusing on the treatment of hypertension, and in high-risk patients, e.g., those with a high risk of stroke or CAD, low-dose aspirin such as 75 mg/day can be considered [11].

### 5.1. Treatment of Heterozygous FH

In patients with heterozygous variant FH, a healthy lifestyle is recommended; however, it is not sufficient for the required reduction in serum LDL-C levels and hypolipidemic treatment is necessary. As discussed, the main group of drugs are statins among which high-intensity statins, such as atorvastatin at a dose of 40–80 mg daily or rosuvastatin at a dose of 20–40 mg daily, are used [12]. Stein et al. [39] compared the efficacy of atorvastatin and rosuvastatin in lowering LDL-C fractions during an 18 week study among patients with HeFH and noted that patients treated with rosuvastatin showed a significantly greater reduction in LDL-C levels (57.9%) compared to the atorvastatin-treated group (50.4%) at all doses evaluated (20, 40, and 80 mg). A similar relationship was observed for other cholesterol fractions, showing better results in the reduction of TC, apoB levels, and an increase in HDL-C levels [39]. The meta-analysis by Vuorio et al. [40] also showed that the use of statins in a pediatric population with HeFH significantly lowers LDL-C (−21% to −41%), TC, and raises HDL-C compared to placebo groups [40].

There is also the possibility of ezetimibe monotherapy if there are contraindications or intolerance to statins [1]. Such statin monotherapy is capable of lowering LDL-C levels up to 60% in this group of patients; however, most patients require intensification of therapy by adding another drug, ezetimibe [1,12]. It is suggested that such therapy is capable of lowering LDL-C levels by another 20% [12]. A phase three study evaluating the efficacy of ezetimibe 10 mg and bempedic acid in patients with HeFH using the highest tolerated doses of statins showed that after 12 weeks of combination therapy with these drugs, the highest reduction in LDL-C was demonstrated compared to the placebo group (by 38%), and the groups with ezetimibe or bempedic acid are included. In addition, this group had the highest percentage of patients with LDL-C levels < 100 mg/dL or 70 mg/dL, and the highest LDL-C reduction of up to 50% since the start of the study compared to the other groups. It seems that due to the different mechanism of action, the combination of ezetimibe and bempedic acid leads to a greater reduction in LDL-C than either drug alone [41]. PCSK9 inhibitors are monoclonal antibodies used in the next round if target LDL-C values have not been achieved. Among the drugs targeting PCSK9, we can distinguish alirocumab, evolocumab, and inclisiran. The first two are monoclonal antibodies while inclisiran is a small interfering ribonucleic acid (siRNA). They are drugs administered subcutaneously. They are very effective in lowering LDL-C, reducing the risk of ASCVD, and presenting few side effects—they are a safe treatment. Usually, once they are included, the treatment goal is achieved as they lead to a reduction in LDL-C by another 50–60% [12]. The meta-analysis by Brandts et al. [42] focused on evaluating the effects of treatment with various PCSK9-targeted drugs including alirocumab, evolocumab, and inclisiran in HeFH patients inadequately treated with statins or statins and ezetimibe. Each of these drugs was shown to produce similar LDL-C reductions of up to 50%, regardless of the genetic variant of the disease [42]. Similar results were noted by Ge et al. [43] who showed that PCSK9 inhibitors were able to decrease LDL-C levels significantly (by −49.14%) and TC, TG, non-HDL, apoB, Lp(a), and increase HDL-C and apolipoprotein A1 levels. As mentioned above, it seems that long-term hypolipemic treatment with PCSK9 inhibitors, including alirocumab and evolocumab, is not only effective, but also safe and well tolerated by FH patients [43]. Furthermore, Burnett et al. [44] conducted a meta-analysis examining the efficacy of various adjunct therapies in patients with FH who are already using the highest possible doses of statins and are at increased cardiovascular risk. Evolocumab, alirocumab, and inclisiran were shown to have greater efficacy in further lowering LDL-C compared to placebo, ezetimine, and bempedic acid [44]. In more severe cases, adjunctive treatment with lipoprotein apheresis (LA) is possible [23]. Among others, it is recommended for the patients in question with LDL-C levels > 300 mg/dL, or for those with additional high cardiovascular risk with accompanying LDL-C levels > 200 mg/dL, or patients with diabetes/CVD and LDL-C levels > 160 mg/dL [45]. Raina et al. [46] conducted a study in which they evaluated the role of LA as a next-line treatment option for lowering LDL-C levels. LA therapy as a subsequent treatment option appears to have significant efficacy in reducing LDL-C and Lp(a) levels and prevents the development and worsening of CVD in FH patients whose lipid levels are inadequately lowered with available pharmacotherapy [46].

### 5.2. Treatment of Homozygous FH

The homozygous form of FH is much less common than the heterozygous form, while it is characterized by much higher serum LDL-C levels in childhood and has a higher risk of atherosclerosis and cardiovascular events, and, therefore, requires aggressive treatment [10]. Patients should be treated in specialized centers with a multidisciplinary team providing expert patient supervision [1]. Among these patients, initial treatment looks exactly like that of the HeFH group, but therapy with statins and ezetimibe does not lead to a significant reduction in LDL-C levels, so intensification of therapy with PCSK9 inhibitors is necessary. However, the response to this treatment is dependent on the type of gene defect, and better results were achieved in the group with a defect in the LDL-C receptor and any LDLR function present [12,47]. Contrary to patients with HeFH, triple-drug therapy consisting of statin, ezetimibe, and PCSK9 inhibitors is not sufficient. Drugs, such as apoB-100 antisense oligonucleotides (ASOs) and microsomal triglyceride transfer protein (MTP) inhibitors, can be used to further lower LDL-C levels. A drug in the MTP group is lopitamide, which reduces chylomicrons and Very-Low-Density Lipoprotein (VLDL), resulting in a significant reduction in LDL-C levels of up to 50%; however, it is characterized by causing numerous side effects including hepatotoxicity, making its use limited [12]. Nohara et al. [48] in a prolonged study demonstrated that long-term use of lopitamide by patients ineffectively treated with hypolipemic therapy is not only safe, but also effective in further lowering lipid levels, with particular emphasis on the LDL-C fraction. Five Japanese patients continued treatment with lopitamide after the phase three study, and efficacy results were compiled after 60 weeks. After this time, a percentage reduction in LDL-C levels of −35.58% from baseline was recorded, maintaining LDL-C levels < 100 mg/dL. In addition, the treatment in question resulted in a reduction in TC, non-HDL-C, TG concentrations and an increase in HDL-C levels from the start of the study to 60 weeks after phase three [48]. The action of ASO is to stop the production of apo-B, reducing the production of VLDL, thus significantly reducing serum LDL-C levels. A new drug that has applications in lowering LDL-C levels among patients suffering from the homozygous form of FH is the human monoclonal antibody that inhibits angiopoietin 3, evinacumab. This intravenously administered agent has been shown to reduce apo-B-containing lipoproteins by about 50% compared to baseline values; in addition, it rarely causes side effects and is well tolerated [12]. The adjunctive treatments discussed above may show additional benefits because their LDL-lowering effects do not depend on LDLR expression [47]. When pharmacotherapy is unable to achieve target LDL-C levels, LA is another therapeutic option [1,12]. It involves the removal of apo-B lipoproteins from plasma and is intended for patients with LDL levels above 300 mg/dL [12,45]. Treatments last from 2 h to 4 h, usually taking place for up to a week in a specialized center [47]. Another therapeutic option is liver transplantation, as the new organ contains numerous LDL receptors, thus leading to a significant decrease in serum LDL-C levels [1,23].

**Figure 3:**
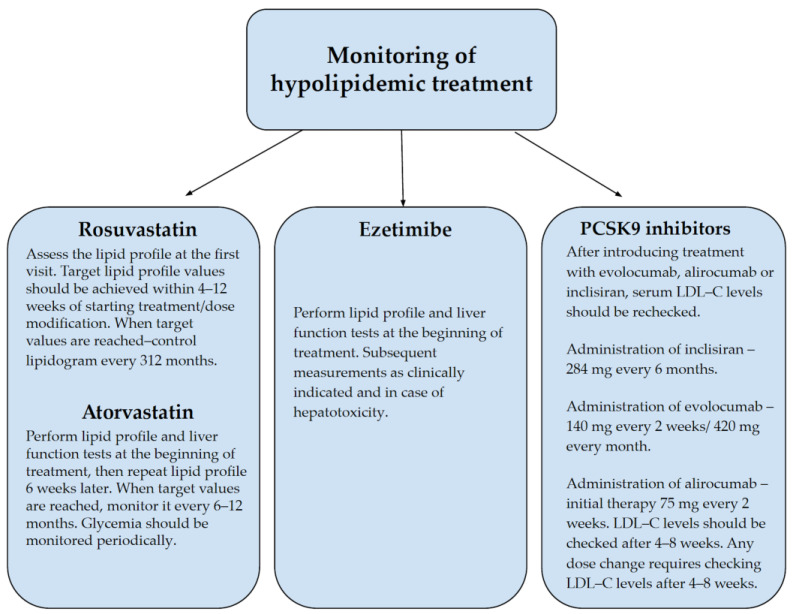
Basic Monitoring Data for Hypolipidemic Treatment [49,50,51,52].

During pharmacotherapy, LDL-C levels should be monitored regularly, preferably every 2–3 months, to modify treatment to achieve the desired therapeutic effect [23]. Figure 3 shows the basic monitoring data for hypolipidemic treatment [49,50,51,52].

## 6. Cardiovascular Risk and Complications in Patients with FH

CVD are the most common cause of premature deaths worldwide [53,54,55]. They account for 49% of mortality in Europe. According to estimates, CVD will contribute to approximately 23.6 million deaths per year by 2030 [55]. This makes CVD a heavy burden on health care [54] consuming significant financial resources [55]. Assessment of CVD risk factors is crucial for their effective control and treatment [53,54,55]. The result of failure to properly diagnose and treat CVD can be sudden death [55]. Although elderly people have the highest risk of developing CVD [54], for some diseases, serious cardiovascular complications can also develop in children and the young, as in the case of FH [5]. In addition, age is an essential non-modifiable CVD risk factor in HeFH patients [56].

In FH patients, CVD risk is fundamentally influenced by genetic factors [57], which are described in detail in paragraph two. Depending on the homozygous or heterozygous form of FH, and thus the degree to which the metabolism of LDL-C particles has been disrupted, the clinical picture of patients varies in terms of the severity of disease symptoms and the age at which the first signs of CVD occur [10,23]. The disease leads to increased and premature atherosclerosis, primarily the deposition of atherosclerotic plaques in the proximal aorta and coronary arteries [11]. Following increased concentrations of LDL-C, there is its uptake by scavenger receptors and accumulation in macrophages, transforming them into foam cells, thus initiating atherosclerotic plaque formation [10].

A study by Trinder et al. [58] analyzed the DNA of 626 patients with FH. They investigated the effect of the genetic basis of FH (monogenic or polygenic) on the development of CVD events before age 55. They proved that the presence of a monogenic variant correlates with increased CVD risk. However, this relationship has not been shown for the polygenic cause of FH, which is associated with the same risk of CVD events as in patients without a specific genetic cause of the disease. Interestingly, the coexistence of a monogenic variant of FH with an increased polygenic risk score is associated with the highest risk. An analysis of 48,741 patients in the Trinder et al. [59] cohort study provides convergent findings. However, a polygenic cause of FH has been shown to correlate with an increased risk of CVD events compared to patients without a specific genetic cause of FH [59]. Hence, genetic testing is an important component in predicting CVD events [58,59].

Due to its limitations, tools for predicting ASCVD occurrence, such as the American College of Cardiology/American Heart Association (ACC/AHA) ASCVD risk calculator, should be used with caution in patients with FH. In addition to age and race restrictions, this tool is not suitable for assessing ASCVD risk when TC > 320 mg/dL [57]. Paquette et al. [60] reports that the FH-Risk-Score has been developed, which can significantly contribute to individual CVD risk assessment and enable more effective treatments of FH. In addition, the FH-Risk-Score allows for the assessment of future ASCVD occurrence and prediction of death from CVD causes [60]. There have been ideas to incorporate inflammatory biomarkers into CVD risk prediction; however, none has yet met adequate conditions in terms of sensitivity and specificity. Further studies are needed [61]. The effect of a cholesterol-lowering diet, supplemental sterols, and plant stanols on CVD risk in FH patients has also not been proven. However, it is known that the cholesterol-lowering effect in FH patients occurs with the addition of sterols and plant stanols, while omega-3 fatty acids lower TG [62].

The consequence of constant exposure to increased LDL-C levels is the occurrence of MI about 20 years earlier, compared to the general population [63]. In HoFH, cholesterol accumulation occurs as early as childhood, resulting in symptomatic valvular stenosis [5,9]. Untreated disease is associated with a higher risk of MI, angina, peripheral artery disease (PAD), or stroke [11]. Treatment does not protect against the development of atherosclerosis [3], while in the absence of treatment, death occurs in patients < 20 years old [5,9]. The most common causes of death are MI, angina, and aortic supravalvular or valvular stenosis [3]. Table 3 shows examples of complications of FH [23,63].

The purpose of the meta-analysis by Akioyamen et al. [64] was to determine the association between the HeFH phenotype and ischemic stroke and PAD. After verification, six studies involving 183,388 participants with a mean age of 44 to 59 years were included in the meta-analysis. After reviewing the studies, it is suggested that the heterozygous form of FH is associated with an increased risk of PAD and stroke; however, in patients with genetically confirmed disease, its association with stroke is uncertain, thus further studies are needed [64]. In contrast, Farnier et al. [65] conducted a clinical trial in which they assessed the prevalence of HeFH in patients admitted to a coronary care unit for acute MI and the severity of CAD. Patients were evaluated according to DLCN criteria for the presence of FH. It was found that among patients with acute MI, 2% of them had FH, which is five times more people than expected in the general population. They were young, with inadequate hypolipidemic therapy, characterized by severity of CAD disease confirmed by the SYNTAX scale, more frequent hypertension, and PAD. It is noteworthy that FH patients who have had an incident MI or ACS have a worse prognosis, as they have an approximately two times higher risk of recurrence of these or other cardiovascular events [65]. Alothman et al. [66] were interested in the topic of quality of life in patients with HoFH. Patients were assessed using Health Related Quality of Life and observed a deterioration in disease-related quality of life in the form of lower scores in social functioning or general health, among other things. This is an interesting topic that needs to be studied in more depth [66].

## 7. Evinacumab Mechanism of Action

Evinacumab’s action mechanism is multilevel and affects lipid concentrations through various pathways [67]. First, this involves inhibition of ANGPTL3 activity leading to lack of inhibition of individual lipase activity [16,67,68]. The reduction in TG concentrations is due to the lack of LPL inhibition, which enables lipoprotein hydrolysis [67]. Studies directed at the ANGPTL3 protein have also shown improved glucose and insulin tolerance, combined with a reduction in stored TG in the liver [69]. Another pathway leading to the lowering of another lipoprotein, HDL, is the lack of inhibition of EL. Through this blockade, free hydrolysis of HDL phospholipids is possible [67,68,70]. Additionally, it is worth mentioning how the inhibitory effect on ANGPTL3 affects apoB. Evinacumab causes an increase in the catabolism of apoB intermediate density lipoprotein (IDL) and apoB LDL. Consequently, there is an increase in apoB clearance and a decrease in its plasma concentration [19]. The last and least understood mechanism is the one responsible for the decrease in LDL-C concentration with the supply of the monoclonal antibody in question. One study from last year proved that increased EL activity leads to remodeling of VLDL, which are precursors of LDL, resulting in lower LDL-C levels. It should be remembered that in patients with HoFH, the decrease in LDL-C is completely independent of the presence of the LDL receptor [68]. At this point, it is worth pausing for a moment to look more closely at ANGPTL3, the protein that the drug we are discussing blocks, and whose expression occurs in the liver hence it is a hepatokine [71]. The ANGPTL3 glycoprotein has been discovered in serum in two forms—as a full-length protein and as a cleaved, N-terminal ANGPTL3 molecule. Cleaved by furin, the truncated form of the protein is the main component for EL inhibition [22]. The protein in the body undergoes a number of biochemical processes, such as glycosylation and protein cleavage, until it is finally activated. The C-terminal domain mentioned in Figure 1 is responsible for angiogenesis, while the N-terminal end is responsible for inhibiting LPL, EL, and HL activity. In vitro, it has been proven that the reduction in serum VLDL concentration is due to a decrease in VLDL biosynthesis through inhibition of ANGPTL3 [71]. Some human genetic studies show that patients carrying loss-of-function in ANGPTL3 have both lower serum LDL-C, HDL-C, and TG levels. A correlation between variation in lipid levels in carriers of ANGPTL3 loss-of-function variants has also been shown to reduce the odds of ASCVD by about 40% [22,72]. Lower odds of myocardial infarction have also been reported in these individuals. The DiscovEHR study involved whole exome sequencing of 58,335 Europeans. Genotyping identified individuals with loss of ANGTPL3 function. They had half the ANGPTL3 levels of other subjects and a 39% lower probability of coronary artery disease. The genetics and mechanism of ANGPTL3 inhibition clearly illustrate the multiple pathways of lipoprotein lowering and coronary disease risk reduction associated with increasing genetic variants of LPL [67].

## 8. Indications and Contraindications of Evinacumab

Evinacumab is a recommended drug in patients with HoFH [14,17,68,73]. Patients receive this drug as an adjunct to existing lipid-lowering therapies, for example: statins, ezetimibe, and PCSK9 [17,68]. To receive this type of therapy, the patient must be at least 12 years old. Additional diseases qualifying for the supply of evinacumab are familial or non-familial refractory hypercholesterolemia and hypertriglyceridemia representing the severe form [74]. The recommended dose has been set at 15 mg/kg iv. The drug is administered by infusion over one hour once every four weeks [14,16]. According to current medical knowledge, no interactions with other drugs have been observed [68]. The half-life of evinacumab and its elimination is not constant and depends primarily on its concentration in blood. The drug shows high safety in patients with both hepatic and renal insufficiency, due to the lack of effect on the hepatic and renal clearance pathways resulting in no need for dose reduction [14].

A contraindication that was discovered during animal studies is the inability to use evinacumab in pregnant women. Damage to fetuses has occurred among pregnant rabbits, so treatment should be discontinued a minimum of five months before planned conception [14,68]. Thus, patients using evinacumab who are sexually active should mandatorily use effective contraception to negate the likelihood of pregnancy and the appearance of a potential fetal defect [68].

## 9. Side Effects of Evinacumab

The safety of evinacumab was comparable to that of the placebo in both intravenous and subcutaneous forms. In the study, the incidence of adverse effects after a dose of 15 mg/kg iv every four weeks was comparable in Caucasian and Japanese subjects, while at a 3-fold dose reduction, i.e., 5 mg/kg iv every four weeks, the incidence was significantly higher in Caucasians. At 300 mg single-dose group subcutaneously, the Caucasian and Japanese races had similar frequencies of side effects, while at 300 mg every week, the frequency was again higher in the Caucasian race [75]. Adverse effects represent a small percentage of the patients studied so far, with no patients discontinuing treatment, no cardiovascular emergencies, and no deaths [13,67,76]. The most common adverse reactions include headache, flu-like symptoms, dizziness, nasopharyngitis, nasal discharge, nausea, and pruritus at the infusion site [16,18,67,73]. Patients have also been observed to have a transient, up to 3-fold, increase in the upper limit of normal liver enzymes: alanine aminotransferase and aspartate aminotransferase, and increase in creatinine phosphokinase [76]. Incidental cases involved the occurrence of suicide attempt, urosepsis, and anaphylactic reaction [13,68].

## 10. Effect of Evinacumab on Cardiovascular Risk in Patients with HoFH

**Figure 4:**
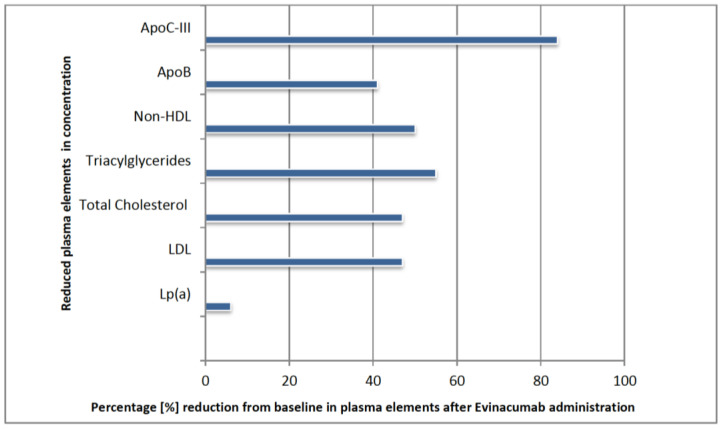
Changes in Plasma Components Affected by Evinacumab after 24 Weeks [16]. ApoC-III, Apolipoprotein C-III; Lp(a), Lipoprotein(a).

Evinacumab’s action mechanism of inhibiting the ANGPTL3 glycoprotein contributes to the reduction of LDL cholesterol, thereby significantly reducing the risk of cardiovascular events [13]. The benefits of the drug, outweigh the fact that it simultaneously reduces HDL cholesterol, which is the desired lipoprotein. To offset this effect, patients can undergo treatment with fibrates, which affect LDL receptors that HoFH patients are lack of [76]. Patients with FH have a significantly higher risk of atherosclerosis and ischemic heart disease compared to the general population [14]. Studies suggest that ANGPTL3 protein may be considered as a marker of cardiovascular events or risk in the future [16]. The evinacumab Lipid Studies in Patients with Homozygous Familial Hypercholesterolemia (ELIPSE HoFH) study was conducted in 11 countries. Participants were diagnosed with HoFH and used pharmacological treatment with a variety of drugs, for example: statins 94% of participants, ezetimibe 75%, PCSK9 inhibitors 77%, lomitapide 22%, or apheresis 34% [76]. The effect of evinacumab was summarized after 24 weeks of use [14,76,77]. There was a reduction in LDL-C of about 47% from baseline values. The change in other parameters is shown in the Figure 4 below [16].

The protective effect in the cardiovascular depiction begins with a decrease in LDL-C levels, which patients can expect as early as 14 days after initiation [14,16]. The decrease in LDL-C trended downward throughout and was independent of concurrent hypolipemic treatment [14]. Evinacumab, as a new treatment for HoFH patients, is a very promising drug that, by reducing cardiovascular risk, reduces mortality in these patients without negatively affecting quality of life [78].

## 11. Effect of Evinacumab in the Treatment of Patients with Lipid Disorders including Familial Hypercholesterolemia

Despite a considerable number of hypolipemic drugs, the treatment options for patients with FH remain limited. However, evinacumab is able to effectively lower atherogenic lipid levels, thereby reducing cardiovascular risk [79].

The phase two clinical trial by Rosenson et al. [77] evaluated the safety and efficacy of intravenous or subcutaneous evinacumab therapy in patients with or without HeFH treated with maximally tolerated doses of common hypolipidemic drugs. After 16 weeks, a decrease in all atherogenic lipid fractions except Lp(a) was noted after evinacumab intervention in a manner proportional to the doses used, with the greatest results in patients using the highest doses of the drug. It has been shown that using the highest doses of evinacumab, LDL-C fraction values in patients with refractory hypercholesterolemia can decrease by up to another 50%. There was no significant difference in the incidence of serious adverse events between groups treated with intravenous or subcutaneous therapy [77]. Raal et al. [13] also conducted a clinical trial in which they compared the effect of adding intravenous evinacumab to standard therapy in patients with ineffectively treated HoFH. After 24 weeks of intervention, there was a 47.1% reduction in LDL-C fraction compared to baseline, while there was a 1.9% increase in LDL-C levels in the placebo group. In addition, decreases in TC, non-HDL-C, and apoB were observed in the intervention group. It is noteworthy that additional apheresis treatment or no apheresis treatment made no difference in final LDL-C levels, since apheresis did not affect evinacumab plasma levels. Importantly, evinacumab showed a beneficial therapeutic effect regardless of LDL receptor activity [13]. Evinacumab also proved highly effective in reducing TG levels in patients with mixed dyslipidemia. In a clinical trial conducted by Ahmad et al. [79] the use of a single ascending dose induced a reduction in TG levels, showing greater efficacy with increasing dose, but up to a level of 5 mg/kg. The use of higher doses did not induce a greater reduction in TG concentrations. Over the course of the study, a reduction in TG concentrations to a level of about 50 mg/dL was recorded, achieving a reduction of 80%. Interestingly, intravenous delivery of the drug was more effective than subcutaneous [79]. An interesting study was conducted by Reeskap et al. [80] who evaluated not only the ability of evinacumab to lower LDL-C levels, but also the progression of atherosclerotic plaque on coronary computer tomography angiography after 6 months of therapy in two young patients with HoFH. The addition of evinacumab to therapy based on statins, ezetimibe, and LDL apheresis resulted not only in a significant reduction in LDL-C, reaching less than 1 mmol/L after LDL apheresis, but also in an almost complete regression of atherosclerotic plaque. Evidence from this study shows that very intensive evinacumab-based hypolipemic therapy at a young age is able to lead to regression of young atherosclerotic plaques [80].

## 12. Conclusions

Elevated LDL-C values underlie premature ASCVD, and symptoms associated with cholesterol deposition, e.g., tendon xanthomas, xanthelasma, and arcus cornealis. The diagnosis of xanthomas of the extensor tendons in a patient is an important signal for diagnosis in the direction of FH, as it is a pathognomonic sign. It is also worth remembering that in HoFH, the first symptoms appear as early as childhood, while in HeFH, clinical symptoms may not appear for a long time, even decades. The absence of clinical symptoms is not a basis for excluding the diagnosis of FH.

Important elements in the diagnosis of FH are LDL-C levels, the presence of clinical manifestations, and family history. The DLCN, Simon Broome Criteria and MEDPED are most commonly used to diagnose HeFH. On the other hand, criteria for the diagnosis of HoFH have been developed by the EAS and are also included in the ICD-10. During the diagnosis of FH, it is worth remembering to exclude conditions that raise LDL-C levels, such as certain chronic diseases, and medications taken. FH is an underdiagnosed disease. Researchers have attempted to explain what this may be due to and have proposed solutions to help increase the percentage of correctly diagnosed FH patients.

Evinacumab has been classified in the family of hypolipemic drugs, with the main indication in patients with HoFH. This is a special and difficult case of patients who, from the beginning, are at increased cardiovascular risk. Added to this is the ease and speed in the formation of atherosclerotic plaques due to the atherogenic effect of circulating plasma lipoproteins on the blood vessels leading to ASCVD. The rapid timing of the drug’s research, the evaluation of its effects of action on animals and humans, and its approval by global organizations, such as the FDA, and the EU’s positive evaluation, has made it possible to start treatment in patients in need. The drug is a very optimistic addition to previous drug therapies, intensification of which has not had the intended effect. It is also important that the drug has shown few side effects in studies to date, good tolerance by patients, and no interactions with other substances. The small percentage of contraindications occurring mainly concerned possible fetal defects in pregnant women taking the drug. Therefore, the safety profile of evinacumab is relatively high. Additionally, it is interesting that the drug lowers not only LDL-C, but also TG, non-HDL, Apolipoprotein C-III, and apoB, which may provide a wide range of new indications and applications in the future, after appropriate clinical trials. The hope is to be able to expand the indications for the use of this monoclonal antibody even if only in heterozygous familial hypercholesterolemia or mixed hyperlipidemia. The impressive decrease in LDL-C levels may also suggest the future use of this drug in severe cases of metabolic syndrome with overlapping diseases that burden the cardiovascular system, for example, hypertension or diabetes. The weakness of this study is that there is a lack of work describing in more detail the mechanism of action of the evinacumab and its biochemical effects on all plasma elements of interest. The drug being on the market for more than a year, there are not yet enough publications on long-term side effects and impact on the body. There is also a lack of papers on other indications for its use.

## Figures and Tables

**Table 1:** A Brief Description of the Major Mutations Involved in the Development of HoFH [12,23].

The Main Mutations Involved in the Development of HoFH	
LDLR	Mutation causing absence/reduced number of LDL receptors capturing LDL-C
PCSK9	Mutation contributes to overactivity of PCSK9, which causes increased destruction of LDL receptors, inhibiting its return to the cell membrane
ApoB	Mutation causing defect in ligand with subsequent decrease in LDL clearance from plasma

HoFH, Homozygous Familial Hypercholesterolemia; LDLR, Low Density Lipoprotein Receptor; LDL-C, Low-Density Lipoprotein Cholesterol; PCSK9, Proprotein Convertase Subtilisin Kexin Type 9; LDL, Low-Density Lipoprotein; ApoB, Apolipoprotein B.

**Table 2:** Comparison of Familial Hypercholesterolemia Diagnostic Criteria [7,8,26,32,33,34].

Diagnostic Criteria	DLCN	Simon Broome Criteria	MEDPED	FAMCAT	ICD-10 (HeFH)	ICD-10 (HoFH)	EAS
LDL-C	+	+ (or TC)	+	+ (or TC)	+	+	+
Family History	+	+	+	+	+	+	+
Clinical Examination	+	+	−	−	−	+	+
Clinical History	+	−	−	−	−	+	−
DNA Analysis	+	+	−	−	+	+	+
Applies to Diagnose HoFH	−	−	−	−	−	+	+

DLCN, Dutch Lipid Clinic Network; MEDPED, Make Early Diagnosis to Prevent Early Death; FAMCAT, Familial Hypercholesterolemia Case Ascertainment Tool; ICD-10, International Statistical Classification of Diseases and Related Health Problems, 10th Revision; HeFH, Heterozygous Familial Hypercholesterolemia; EAS, European Atherosclerosis Society; TC, total cholesterol.

**Table 3:** Complications Developing in the Course of FH [23,63].

Complications of FH
Transient ischemic attack
Stable coronary artery disease
Fatal and non-fatal myocardial infarction
Stroke
Peripheral artery disease
Congestive heart failure
Cerebrovascular accidents
Cardiovascular death
Aortic stenosis

FH, familial hypercholesterolemia.

## Data Availability

The data used in this article are sourced from materials mentioned in the References section.
